# Paralympic health: emerging trends and innovations

**DOI:** 10.3389/fspor.2026.1746523

**Published:** 2026-04-24

**Authors:** Ciro Winckler, Frederico Ribeito Neto, Rodrigo Rodrigues Gomes Costa, Irineu Loturco, Elke Lima Trigo, Andrea Jacusiel Miranda, Luiz Gustavo Santos

**Affiliations:** 1 Department of Human Movement Sciences, São Paulo Federal University, Santos, Brazil; 2 Paralympic Sports Department, SARAH Network of Rehabilitation Hospitals, Federal University of São Paulo, Brasilia, Brazil; 3 NAR-Nucleus of High Performance in Sport, Federal University of São Paulo, Santos, Brazil; 4 SENAC University Center, São Paulo, Brazil; 5 Brazilian Cycling Confederation, South American Football Confederation, São Paulo, Brazil; 6 Department of Sports Development CPB, Brazilian Paralympic Committee, São Paulo, Brazil

**Keywords:** athlete with disability, health, para sport, paralympic, performance

## Abstract

This article synthesizes recent developments in Paralympic Health, covering not only the conceptual evolution that supports athletes with disabilities in Paralympic sports but also the impact of scientific advances on health care and on the classification system, training, and competition. The Paralympic Games are not only a competitive event but also a driving force behind the development of assistive technology and the generation of knowledge about physical exercise for individuals with disabilities. Sports classification, based on increasing scientific evidence, is being consolidated by applying the impairment concept to three major groups: physical, vision, and intellectual. The most significant development in recent editions of the Games is epidemiological monitoring, which has made it possible to reduce the incidence of health problems. The interdisciplinary development of health based on science is fundamental in guaranteeing opportunity and equity for people with disabilities in Paralympic sports.

## Introduction

The genesis of the Paralympic Movement can be traced back to the Stoke Mandeville Games, organized in 1948 under the guidance of Sir Ludwig Guttmann (1). However, historical accounts suggest that sporting activities involving individuals with disabilities date back to 1792, during the Napoleonic Wars. The earliest recorded instance pertains to cricket matches played at the Greenwich Naval Hospital, where teams comprised of arm and leg amputees competed against each other (2). The first international competition for persons with disabilities took place in Paris, in 1924, the Silent Games, for persons with deafness. Historically, the sports movement for people with disabilities has evolved through three main scenarios: the Deaflympics, exclusively for people with deafness, the Special Olympics, for those with intellectual impairments, and the Paralympic Movement, which is our focus in the current work (1).

In this context, the term “Paralympic” refers specifically to the sports contested at the Summer and Winter Paralympic Games, which represent a subset of para sport, the broader category of sports for persons with disabilities. Therefore, not all para sports are Paralympic sports. In this sense, the use of the prefix “para” in Para sport should be understood not solely as parallel, as it is in the Paralympic movement, but to adopt a broader sense, indicating of alongside, besides, near, resembling, beyond (3). This nuance understanding facilitates more precise conceptual application of concepts and a more efficient intervention in health, given that some aspects of the intervention will be unique and specific. At the same time, more general knowledge of athletes without impairment and sports is applicable and utilized in other contexts.

The Paralympic movement has grown significantly, now ranking as the second-largest multi-sport event in the world (4) (Table 1). The Tokyo 2020 Paralympic Games, held in 2021, featured 4493 athletes, representing 162 countries, including the Refugee Paralympic Team. This edition set a record for the number of female athletes participating, with 1,853 female athletes competing alongside 2,550 male athletes. This represented a notable increase in participation compared to previous Paralympic Games, demonstrating the event's growing global reach and inclusivity. In the same trend, the Beijing 2022 Winter Paralympic Games had a total of 564 athletes, competing from 46 countries, and also achieved a record for female participation, with 138 female athletes and 422 male athletes.

**Table 1:** Participation in paralympic games, events and impairment group.

Year	Summer Games	Athletes	Men	Women	Countries	Sports	Events	Impairment groups
1960	Rome, Italy	319	262	56	21	8	114	SCI
1964	Tokyo, Japan	266	195	71	20	9	143	SCI
1968	Tel Aviv, Israel	773	578	195	28	10	188	SCI
1972	Heidelberg, Germany	921	653	268	41	10	188	SCI
1976	Toronto, Canada	1,274	1,003	270	41	13	448	SCI
1980	Arnhem, Netherlands	1,653	1,225	422	42	13	590	SCI, VI, Amputee
1984	Stoke Mandeville, UK	2,110	1,569	541	54	18	975	SCI
	New York, USA							VI, Amputee, CP, LA
1988	Seoul, South Korea	3,042	2,371	671	60	19	733	SCI, VI, Amputee, CP, LA
1992	Barcelona, Spain	2,999	2,300	699	83	16	489	SCI, VI, Amputee, CP, LA
1996	Atlanta, USA	3,252	2,462	790	103	19	519	SCI, Amputee, CP, LA, II, VI
2000	Sydney, Australia	3,871	2,883	988	123	19	550	SCI, Amputee, CP, LA, II, VI
2004	Athens, Greece	3,749	2,600	1,149	135	19	519	SCI, VI, Amputee, CP, LA
2008	Beijing, China	3,951	2,584	1,367	146	20	472	IMP, IPR, LD, SS, CI, VI
2012	London, United Kingdon	4,245	2,742	1,503	160	20	503	IMP, IPR, LD, SS, CI, VI, II
2016	Rio de Janeiro, Brazil	4,329	2,658	1,671	160	22	528	IMP, IPR, LD, SS, CI, VI, II
2020	Tokyo, Japan	4,393	2,547	1,846	162	22	539	IMP, IPR, LD, SS, CI, VI, II
2024	Paris, France	4,436	2,463	1,973	170	22	549	IMP, IPR, LD, SS, CI, VI, II
2028	Los Angeles, USA					23		IMP, IPR, LD, SS, CI, VI, II
	**Winter games**							
1976	Ornskoldsvik, Sweden	198	161	37	16	2	53	VI, Amputee
1980	Geilo, Norway	299	229	70	18	3	64	SCI, VI, Amputee, CP, LA
1984	Innsbruck, Austria	419	325	94	21	3	107	SCI, VI, Amputee, CP, LA
1988	Innsbruck, Austria	377	300	77	22	4	97	SCI, VI, Amputee, CP, LA
1992	Albertville, France	365	288	77	24	2	79	SCI, VI, Amputee, CP, LA
1994	Lillehammer, Norway	469	379	90	31	4	133	SCI, VI, Amputee, CP, LA
1998	Nagano, Japan	562	440	122	31	4	122	SCI, VI, Amputee, CP, LA
2002	Salt Lake City, USA	415	328	87	36	3	92	SCI, VI, Amputee, CP, LA
2006	Turino, Italy	474	375	99	38	4	58	SCI, VI, Amputee, CP, LA
2010	Vancouver, Canada	502	381	121	44	4	64	IMP, IPR, LD, CI, VI
2014	Sochi, Russia	541	412	129	45	4	72	IMP, IPR, LD, CI, VI
2018	Pyeongchang, South Korea	564	431	133	49	5	80	IMP, IPR, LD, CI, VI
2022	Beijing, China	558	422	136	46	6	78	IMP, IPR, LD, CI, VI
2026	Milano, Italy					6	79	IMP, IPR, LD, CI, VI

Between 1960 and 2007, impairment groups were classified based on disability type. From 2007 onward, classifications follow the International Paralympic Committee (IPC) Classification Code, based on functional criteria. SCI, spinal cord injury; A, amputee; VI, vision impairment; CP, cerebral palsy; LA, *les autres*; II, intellectual impairment; IMP, impaired muscle power; IPR, impaired passive range of movement; LD, limb deficiency and/or limb length difference; SS, short stature; and CI, coordination impairment.

**Figure 1:**
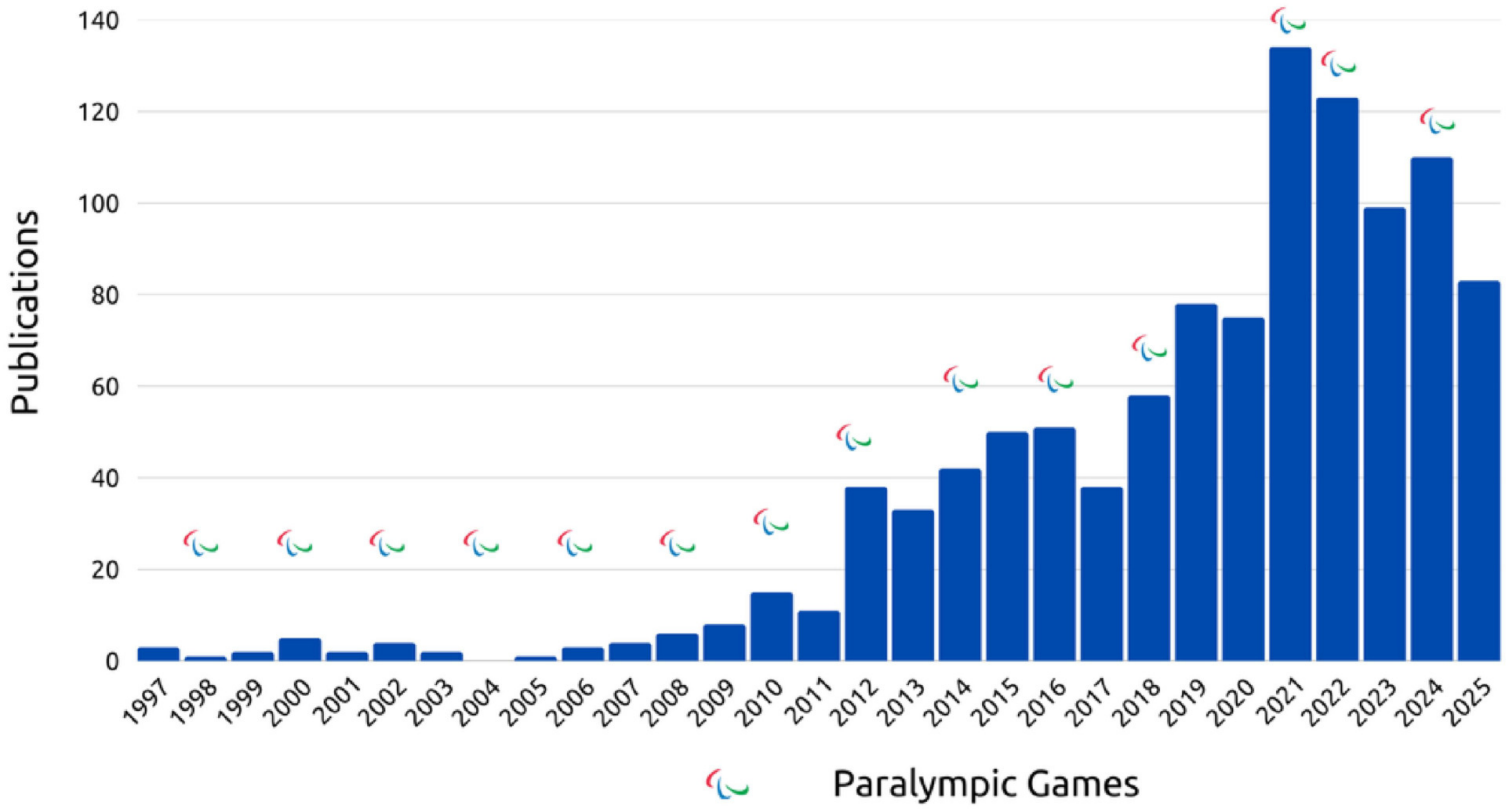
Number of publications on the theme paralympics and health in Web of science across the years. Paralympic games—year of winter or summer paralympic games.

**Figure 2:**
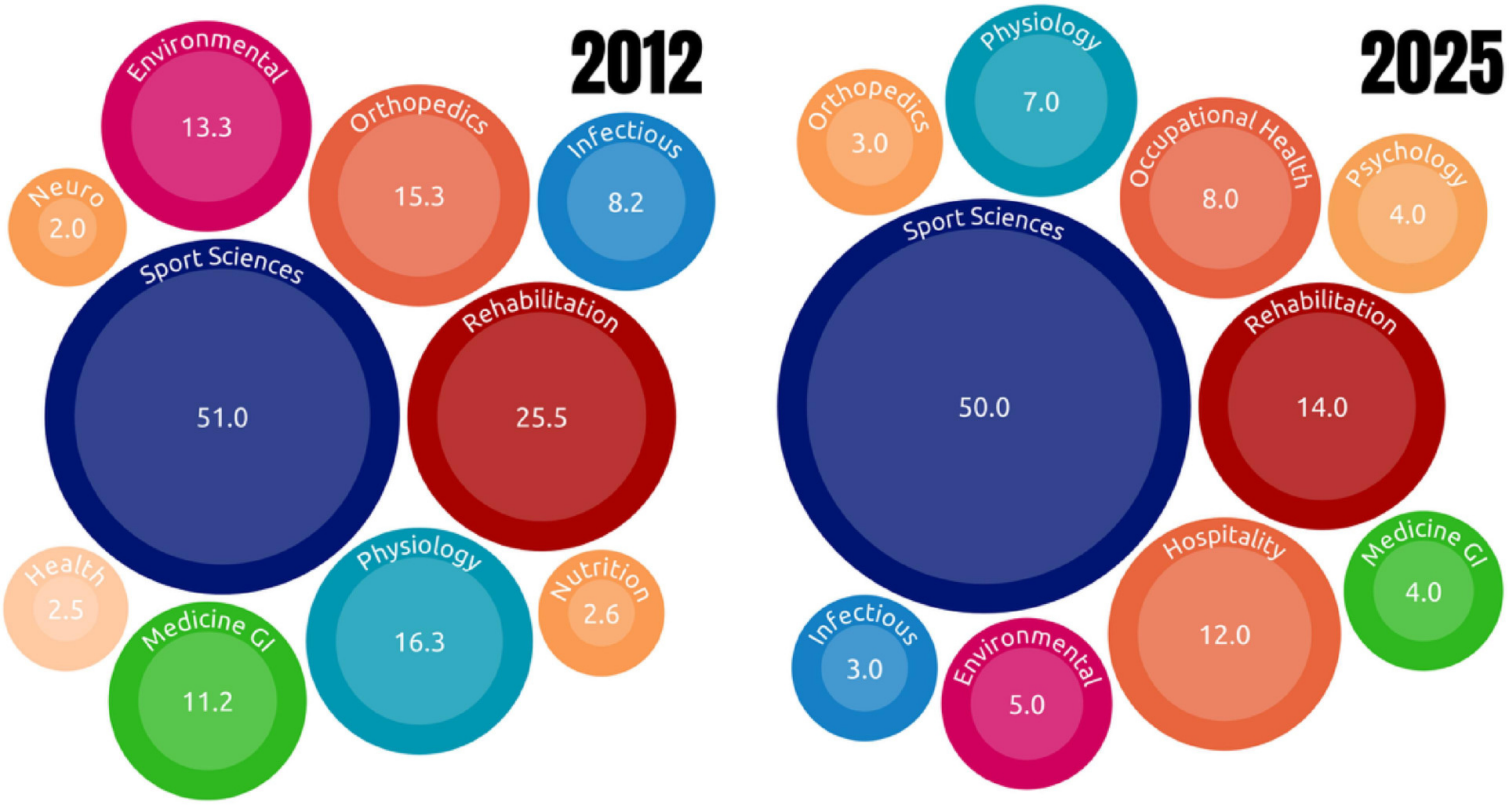
Distribution of publications in web of science categories in 2012 and 2025. Categories are presented as labeled in the figure: sport sciences; rehabilitation—rehabilitation; hospitality—hospitality, leisure, sport, and tourism; cccupational health—public environmental occupational health; physiology; psychology—psychology applied; environmental—environmental sciences; medicine GI—medicine general and internal; infectious—infectious diseases; educational research; multidisciplinary sciences; health—health care sciences and services; nutrition—nutrition and dietetics; orthopedics; neuro—clinical neurology.

In 2012, this esteemed journal published an article by Webborn and de Vliett (5) that sought to address the challenges faced by healthcare professionals in promoting the health of elite athletes with impairments engaged in competitive Para sports. However, the scientific landscape was quite limited, with only 45 publications on the subject of **Health** and **Paralympic** on the Web of Science database (Figure 1). In 2025, the figure of only 1,081 studies in indexed journals underscores the need to revisit and explore new research pathways in this area. During this period from 2012 to 2025, the main areas of study remain sports science and rehabilitation (Figure 2).

In this manuscript, impairment, disability, and health are conceptualized using the International Classification of Functioning, Disability and Health (6). Within this framework, impairment refers to problems in body functions or structures, while disability arises from the interaction among impairments, activity limitations, participation restrictions, and contextual factors. Health is therefore understood as a dynamic, context-dependent construct that extends beyond the underlying impairment and is shaped by sport-specific demands, training exposure, environmental conditions, and the use of assistive technology.

Guided by this conceptual perspective, the present manuscript adopts a narrative and integrative review approach. The literature was selected for its relevance to Paralympic sport, scientific impact, and contribution to understanding health-related issues across impairment groups and high-performance contexts. Rather than aiming for exhaustive coverage, the review prioritizes domains that have shown substantial development over the last decade or that pose specific challenges to athlete health in Paralympic sport. Accordingly, the manuscript is organized to progress from impairment-based frameworks and classification to health outcomes, injury and illness epidemiology, technological innovations, and medication-related considerations, providing a coherent and practice-oriented synthesis (6).

## Paralympic sports

Forty sports have been included as part of the program over the twenty-nine editions of the Paralympic Games (16 summer and 13 winter). Currently, there are 23 summer sports and 6 winter sports (Table 2).

**Table 2:** Sport of summer and winter paralympic games.

Summer Sport	Impairment Group	Governing body	Inclusion Date
Blind Football	VI	IBSA	2004
Boccia	IMP, IPR, LD, CI	BISFed	1984
Cycling Road	IMP, IPR, LD, CI, VI	UCI	1984
Cycling Track	IMP, IPR, LD, CI, VI	UCI	1996
Goalball	VI	IBSA	1976
Para Archery	IMP, IPR, LD, SS, CI	WA	1960
Para Athletics	IMP, IPR, LD, SS, CI, VI, II	WPA	1960
Para Badminton	IMP, IPR, LD, SS, CI	BWF	2020
Para Canoe	IMP, IPR, LD, CI	ICF	2016
Para Climbing	IMP, IPR, LD, SS, CI	IFSC	2028
Para Equestrian	IMP, IPR, LD, SS, CI, VI	FEI	1984
Para Judo	VI	IBSA	1988
Para Powerlifting	IMP, IPR, LD, SS, CI	WPP	1964
Para Rowing	IMP, IPR, LD, CI, VI	FISA	2008
Para Swimming	IMP, IPR, LD, SS, CI, VI, II	WPS	1960
Para Table Tennis	IMP, IPR, LD, SS, CI, II	ITTF	1960
Para Taekwondo	IMP, IPR, LD, CI	ITF	2020
Para Triathlon	IMP, IPR, LD, CI, VI	WT	2016
Shooting Para Sport	IMP, IPR, LD, CI	WSP	1976
Sitting Volleyball	IMP, IPR, LD, CI	WPV	1976
Wheelchair Basketball	IMP, IPR, LD, CI	IWBF	1960
Wheelchair Fencing	IMP, IPR, LD, CI	WAS	1960
Wheelchair Rugby	IMP, IPR, LD, CI	WWR	2000
Wheelchair Tennis	IMP, IPR, LD, CI	ITF	1992
**Winter Sport**			
Para Alpine Skiing	IMP, IPR, LD, CI, VI	FIS	1976
Para Biathlon	IMP, IPR, LD, CI, VI	FIS	1988
Para Cross Country	IMP, IPR, LD, CI, VI	FIS	1984
Para Ice Hockey	IMP, IPR, LD, CI	WPIH	1976
Para Snowboard	IMP, IPR, LD, CI	FIS	2018
Wheelchair Curling	IMP, IPR, LD, CI	WC	2006

BISFed, boccia international sports federation; BWF, badminton world federation; FEI, fédération equestre internationale; FIS, international ski and snowboard federation; FISA, fédération internationale des sociétés d'aviron; IBSA, international blind sports federation; ICF, international canoe federation; IFSC, international federation of sport climbing; IPC, international paralympic committee; ITF, international tennis federation; ITTF, international table tennis federation; IWBF, international wheelchair basketball federation; UCI, union cycliste internationale; WA, world archery; WAS, world abilitysport; WC, world curling; WPA, world para athletics; WPIH, world pIce hockey; WPP, world para powerlifting; WPS, world para swimming; WPV, world para volley; WSP, world shooting para sport; WT, world triathlon; WWR, world wheelchair rugby (7).

Paralympic sports are structured in such a way that some are designed for a specific impairment group (e.g., Para canoe), while others encompass multiple eligible impairments (e.g., Para athletics). This structure influences the dynamics of the game, its rules, and the sports classification system for athletes.

## Impairment groups

Athletes are required to undergo assessment to determine their eligibility for Paralympic sport through a sport-specific classification system. In this context, impairment refers to relatively stable characteristics of body functions or structures that are relevant for classification and competition eligibility, rather than to health status *per se*. Health-related issues discussed in the following sections are therefore not inherent consequences of impairment, but emerge from the interaction between impairment, sport-specific demands, training exposure, and environmental conditions. This sportive classification has evolved profoundly since its inception. Initially, it heavily relied on medical evaluations, which failed to adequately consider the impact of injuries on athletic performance. However, in the 1980s and 1990s, many coaches, athletes, and classifiers recognized this limitation, leading to the development of a functional classification system (8).

In the 2000s, the International Paralympic Committee (IPC) recognized the need for a more standardized approach and introduced of the Classification Code. This document has been refined through various versions to establish which disabilities are eligible for Paralympic sport, setting the premise of classification based on scientific evidence and the characteristics of each sport (8).

The 2024 Classification Code (8) provides a structured framework for the Paralympic Movement, aiming to unify terminology and eligibility criteria for impairments, thereby creating fairer conditions for Para sports. In essence, there has been a shift from medical to functional classification, with the current sports classification system now grounded in scientific evidence. However, some sports still base their classification models on medical assessments for sports classification, such as those for athletes with vision impairment and archery.

The current regulations structure Impairments into three main eligibility groups: physical, vision, and intellectual. Physical impairments are further classified into five subgroups: impaired muscle power, impaired passive range of movement, limb deficiency and/or limb length difference, short stature, and coordination impairment (hypertonia, ataxia, and athetosis) (8).

## Paralympic health issues

From a biopsychosocial perspective (9), health risks in Paralympic sport are not inherent to impairment itself but emerge from its interaction with sport-specific demands, training exposure, and environmental constraints. While Webborn and Van de Vliet (5) highlight that many athletes with disabilities present pre-existing medical conditions that may increase vulnerability to illness, these risks vary substantially according to diagnosis, sport characteristics, and the origin and timing of impairment. For instance, spinal cord injury may be associated with specific secondary conditions such as autonomic dysreflexia or recurrent urinary tract infections, whereas different Para sports accommodate varying impairment severities and support needs. Accordingly, the following sections present the main impairment groups eligible for Paralympic sport, focusing on their predominant etiologies and the health-related implications most relevant to training and competition contexts (10).

## Vision impairment (VI)

Athletes are required to undergo an assessment to determine their eligibility for Paralympic sport using a sport-specific classification system. In this context, impairment refers to relatively stable characteristics of body functions or structures that are relevant to classification and competition eligibility, rather than to health status *per se*. The health-related issues discussed in the following sections are therefore not inherent consequences of impairment itself, but arise from the interaction between impairment, sport-specific demands, training exposure, and environmental conditions (8). The etiology of a disability can significantly impact an athlete's experience in sports. An epidemiological study in Paralympic sports found that 38.9% of the causes of VI are due to retinal-related pathologies (11). For instance, athletes with low vision due to retinal detachment may face challenges with jump training or high-intensity exercise, as jarring movements could exacerbate their condition. Additionally, athletes may be experiencing Charles Bonnet Syndrome (12), a condition characterized by complex visual hallucinations in individuals with no cognitive impairment and may encounter unique difficulties due to the unpredictable nature of their visual disturbances.

Lastly, light deprivation alters circadian rhythm variation, which interferes with sleep patterns and vigilance, thereby influencing the physical performance of athletes with VI (13). This variation also affects hormonal cycles (14). In this scenario, one relevant clinical concern is the prevalence of depression in this population (15).

## Intellectual impairment (II)

Intellectual impairment is defined as a condition that limits intellectual functioning and adaptive behavior relevant to sport performance and classification. While intellectual impairment is not intrinsically a health disorder, athletes with this impairment may present higher vulnerability to certain mental health conditions and medication use, which can influence training tolerance, decision-making, and competitive performance (8). This condition impacts environmental readings, decision-making (e.g., technical and tactical) (16), and social relationships. Anatomical and physiological variations only occur primarily in athletes with syndromic conditions (e.g., Down's syndrome), which alter muscular, circulatory, and ligament stability and hormonal patterns, leading to significant changes in performance (17). Anxiety disorders, depression, and attention deficit hyperactivity disorder are highly prevalent in this population (18).

## Impaired muscle power (IMP)

Athletes with IMP have a health condition that either reduces or makes it impossible for them to voluntarily contract their muscles to move or produce force (8). This impairment includes health conditions such as *spinal cord injury*, *spina bifida*, and *Poliomyelitis*.

*Spinal cord injury* (SCI) involves distinct characteristics that impact the monitoring of an athlete with this condition: impaired cardiovascular function, neuropathic bladder and bowel dysfunction, thermoregulatory responses, skin breakdown, and pressure sores. The primary concern is compromised cardiovascular function in individuals with SCI, which includes challenges in regulating sympathetic vasoconstriction, controlling heart rate, and maintaining adequate cardiac output (19). These challenges emphasize the importance of exploring alternative approaches for monitoring training intensity beyond just relying on heart rate. Recent research has shown that prescribing exercise intensity for adults with SCI does not lead to a uniform distribution across intensity domains (20). Given the individual variability, it is proposed that prescriptions be individualized to reduce variability. Secondly, SCI individuals frequently experience neuropathic bladder and bowel dysfunction, requiring management through medications, catheterization, and, sometimes, other invasive procedures to ensure proper bladder and bowel emptying (21). These characteristics demand attention before training or competition to avoid autonomic dysreflexia associated with failure to empty the bowel and bladder.

Moreover, the third aspect is the impaired thermoregulation that individuals with SCI display in response to increased core temperatures, with increased injury years potentially enhancing chest sweating to compensate for reduced cutaneous vasodilation (22). Therefore, exercising in cool and hot conditions requires special attention. Implementing cooling techniques can mitigate the risk of heat-related issues, while wearing protective clothing is essential in cold environments (23).

The final facet to consider is the cutaneous deterioration and decubitus ulcers often seen in SCI. These ulcers are characterized by localized tissue damage resulting from prolonged pressure, typically over bony prominences, which can lead to varying degrees of ulceration and potential complications (24). Therefore, personalized education, assessment, and treatment of pressure injuries in SCI patients are crucial for preventing skin breakdown and promoting healing. The support of an interdisciplinary team is essential for monitoring the skin, analyzing adaptations to sports equipment, and devising strategies to prevent skin injuries in this population.

Spina bifida (SB) is a defect in the closure of the vertebral arches (25). SB occulta is covered by skin and is often discovered due to other tissue abnormalities. In individuals with SB aperta (SBA), the spinal cord is exposed or in a herniating sac at birth (26). The term myelomeningocele (MMC) is used for all SBA subtypes, even in cases without a cystic component and in those with an open neural plate (27). SB may present similar changes to SCI, depending on the level of impairment; therefore, it is important to consider the same precautions in relation to SCI. A significant consideration in training Para sports for MMC is that these individuals may present diminished executive functions. Nevertheless, the classification of many athletes with MMC is based solely on physical impairment. This underscores the significance of exercising caution regarding the cognitive aspects during sports activities involving this group.

Another etiology is Poliomyelitis, caused by infection with an enterovirus that attacks motor neurons, mainly those in the spinal cord (28). With the introduction of polio vaccines, extensive and systematic vaccination campaigns successfully curbed the virus's spread. However, according to a 2015 WHO report, poliomyelitis continues to be endemic in Afghanistan and Pakistan. Individuals with a history of poliomyelitis may develop post-polio syndrome (PPS), which can negatively affect physical performance over time. PPS is recognized by the gradual or abrupt onset of progressive and persistent novel muscular weakness or anomalous muscular fatigability (diminished endurance), accompanied or not by generalized fatigue, muscular atrophy, or muscular and articular pain, in addition to new respiratory or swallowing difficulties (29). These factors can influence the training session and performance in Paralympic sports, and depending on the severity and eligibility, may result in a change in sporting class or even a transition to a different Para sport, such as from wheelchair basketball to wheelchair rugby.

## Limb deficiency (LD)

This group includes athletes who have structural limitations for the joints to be moved passively, that affect the structure of bones and joints (impaired passive range of movement), as well as those with total or partial absence of a limb or anatomically irregular dimensions of the limb, resulting from trauma, disease, or congenital causes (8). Amputations due to accidents are a frequent cause of this condition.

The impact of this condition depends on the number of joints and limbs involved, and the person can use orthoses or prostheses to enhance the movement of the remaining structures. Athletes with LD exhibit greater energy expenditure and gait asymmetries, which can lead to joint overload (30)The use of prostheses can serve as an empowering factor, benefiting not only the athletic performance of athletes with LD but also enhancing their overall quality of life (31).

## Short stature (SS)

This condition is characterized by a reduction in total body length as a result of congenital or developmental conditions impacting the bones of the trunk, head, and upper and lower limbs (8). This growth impairment is associated with restricted joint mobility and reduced cardiovascular capacity. There is a high prevalence of neurological symptoms, pain, and, in some cases, hearing, voice, and vision deficits (32).

## Coordination impairment CI

Athletes with CI present movement disorders that affect their ability to produce a full range of skilled movements voluntarily, caused by altered health conditions in the structure and function of the central nervous system. Movement patterns are characterized as hypertonia and/or spasticity, motor ataxia, and dyskinesia (8).

One of these conditions is cerebral palsy, a permanent, non-progressive brain disorder caused by an injury to the developing brain, either before birth, during birth, or shortly after birth. The severity of the condition can vary, and it affects motor function and other areas of development, such as communication, intellectual ability, and epilepsy (33). Paralympic athletes with cerebral palsy exhibit altered pacing strategies, resulting in chronic performance decrements and higher heart rates and higher perceived exertion when compared to athletes without impairments (34).

Acquired brain injuries and stroke constitute forms of brain damage that occur postnatally. The latter is defined as the neurological manifestation of a significant reduction in cerebral blood flow in a circumscribed region of the brain, attributable to the obstruction of a major cerebral artery (35). Conversely, the former results in alterations in normal neuronal tissue activity and/or structure, arising from a traumatic or nontraumatic injury (36). These individuals can participate in sports or engage in vigorous exercise, which represents a viable and promising intervention for young adults experiencing post-acquired brain injury fatigue. This participation can effectively enhance aerobic fitness and foster the capacity for exercise self-management (37). Certain characteristics that might be present in this audience and which could potentially interfere with the conduct of training and the athlete's development in sport include alterations in memory, attention, executive function, language (e.g., aphasia), and hemineglect (ignoring or failing to respond to stimuli delivered on one side of the body or circumambient hemispace, even in the absence of primary sensory or motor loss).

## Injuries and illness in paralympic sports

**Figure 3:**
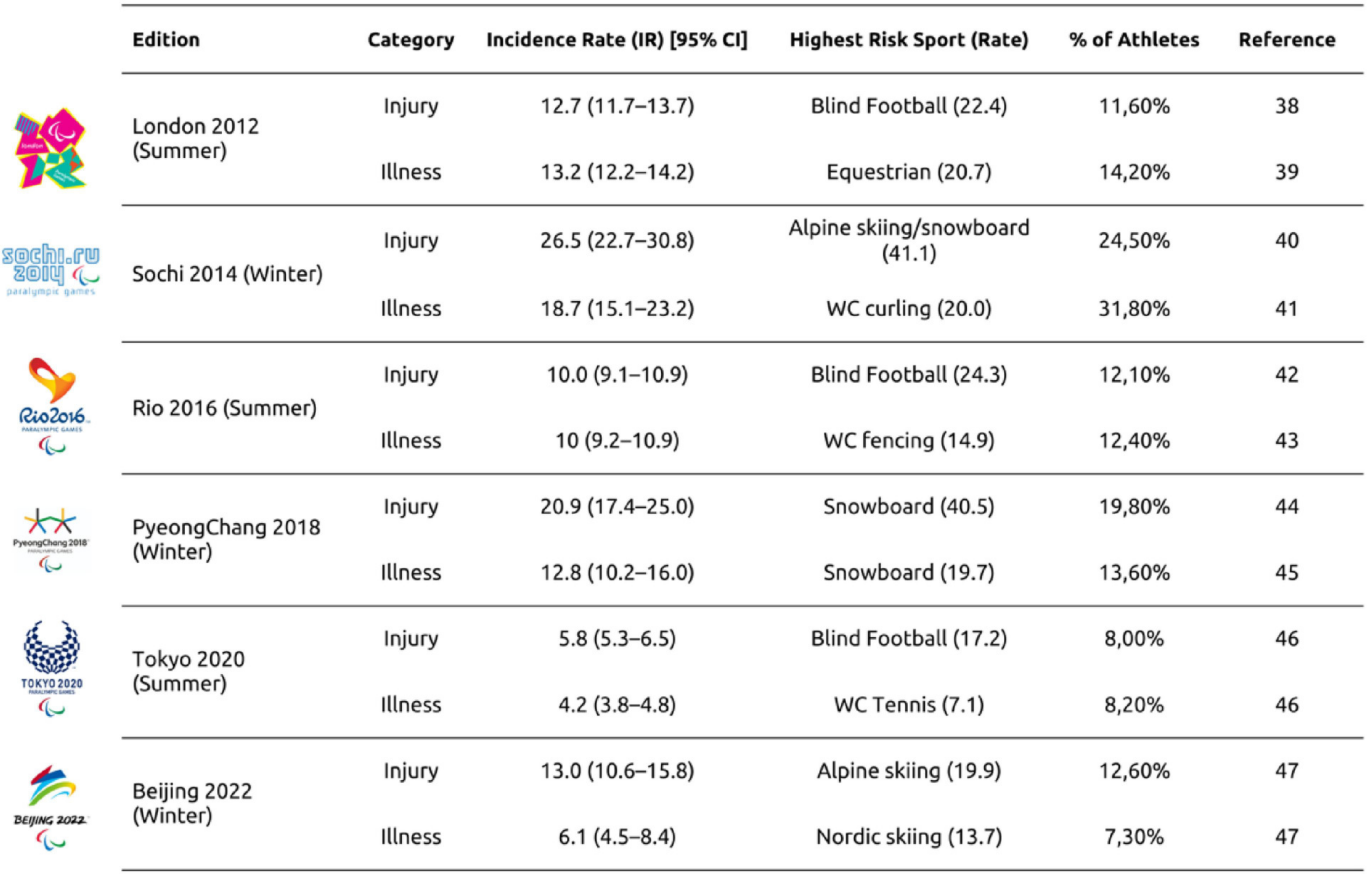
Incidences of injuries and illness in summer and winter paralympic games—incidence rate (IR) = illnesses or injury per 1,000 athlete days (95% CI), sport with the highest incidence and percentage % of the population of the games; references (38–47).

Throughout the Paralympic cycles, studies of injury [any sport-related musculoskeletal or neurological complaint that prompts an athlete to seek medical attention, regardless of time loss from training or competition (38)] and illness [defined as any newly acquired illness or an exacerbation of a pre-existing illness occurring during training or competition, and during or immediately before the Paralympic Games (39)] profiles at the Paralympic Games have informed the optimization of intervention programs and the standardization of preventive actions. These efforts have significantly influenced athletes' training environments and practices, enhancing overall safety and performance. Understanding athletes' functional capabilities, including the use of competition equipment (e.g., wheelchairs, prostheses, or sit-skis) or an orientation system (e.g., athletes with VI in goalball using the sound of the ball and tactile markings on the ground), as well as the athlete's disability, enables accurate interpretation of injury and illness, as shown in Figure 3.

The most frequently recurring illnesses are those affecting the respiratory system, which can be attributed to environmental conditions, such as extreme temperatures (both cold and heat). Additionally, contact injuries may occur due to the use of competitive uniforms or physical contact with equipment or opponents. Gastrointestinal illnesses are also common, often associated with dietary changes or viral and bacterial infections. During the Winter Games, hypothermia and muscle injuries resulting from cold-induced muscle stiffness may occur. In outdoor sports during the Summer Games, heat-related illnesses must be considered given the intensity of the sports and the impact of global warming across different environments, disability conditions and sports (39, 41, 47). This is particularly significant for athletes with SCI, who may have limited thermal control, or athletes with intellectual or vision impairments, who may have limitations in the domain of injuries. Some studies have elucidated the significant role of the inherent nature of Para sports and the specific characteristics of the disability in influencing the incidence and severity of injuries (43, 46–48). In particular, contact sports such as blind football, wheelchair rugby, wheelchair basketball, and taekwondo present unique challenges (46). In addition to the inherent dynamics of the game itself, injuries in these sports are often associated with collisions between athletes and their environment due to limited spatial orientation, particularly in the case of blind football. Furthermore, wheelchair sports exhibit a higher prevalence of upper limb injuries, while taekwondo, in its inaugural appearance at the 2020 Tokyo Paralympic Games, demonstrated a relatively high injury rate (IR) and severity level (46).

Participation in power sports like athletics frequently results in falls and muscle injuries. Tendinitis and stress fractures are common occurrences in endurance sports, such as triathlon and Para-cycling. In technical and precision sports, such as archery and shooting, injuries are primarily associated with improper posture and repetitive movements of large muscle groups.

Research into the incidence and consequences of concussion has become relevant for athletes in winter sports, due to high speeds on snow, and in summer contact sports, especially the aforementioned blind football (48).

In summary, each Para sport tends to have a more substantial impact on the frequency and nature of injuries due to its physical demands and the training and competition environment. Simultaneously, the particularities of each disability can influence the type and severity of injuries, primarily because of the predisposition can creates for certain sport-specific activities. The impact of disability may necessitate specific adaptations in equipment and training strategies to mitigate the risk of injury.

Undoubtedly, understanding these aspects and acting in conjunction with the creation of preventive strategies, which involve rule modifications and educational programs with athletes, coaches, and medical staff, can be crucial factors in reducing the IR and the percentage of athletes affected by injuries and illnesses during Paralympic competitions.

## Equipment and technology

Adapted equipment and technology play a crucial role in promoting social inclusion and enabling daily living tasks for people with disabilities. The progress of Para sports is closely linked to the development of wheelchairs, prostheses, and the advancement in materials and instruments used in each specific sport. The IPC created a regulation with a focus on equipment, guided by four principles: safety, fairness, universality, and physical prowess. According to these regulations, athlete performance should not be achieved through automated, computerized, or robotic equipment (49).

These technological advancements enhance the performance and safety of athletes and promote greater accessibility and inclusivity in Para sports, fostering a more equitable environment for all participants.

Evidence of these advancements includes, for example, custom-made hand orthoses for playing sitting volleyball, designed and manufactured using digital modelling and 3D printing to facilitate accessibility (50). Similarly, another example of technological evolution in adapted equipment is the inquiry by Webborn and Van de Vliet (5) regarding performance enhancements with prostheses and the impact of the socket interface on prostheses and increased loads on the body (5). Recent studies have focused on socket adaptations and fine-tuning of dynamic alignments, further improving the functionality and comfort of athletes using prostheses without compromising their safety (51, 52).

The increasing development of specific adapted equipment and technology for each Para sport is evident. Examples of advancements in research include seated shot-put equipment (53), seating for Para rowing (54), wheelchairs for Para table tennis (55), prostheses for Para-cycling (56) and Para canoe (57), as well as for running athletes with amputations (58). Additionally, configurations of starting blocks for sprint races (59) and enhancements for wheelchair sports performance contribute to this progress (60, 61). These innovations underscore the continuous advances in adapted equipment and technology across various Para sports. Moreover, while Para sports classification is a defining factor for the obtained results, stratifying heterogeneous diagnoses and diseases into similar groups for competitions requires improvements that can be assisted and supported by the gradual development of adapted (i.e., specialized) equipment and technology (62–64).

Finally, it is important to highlight that the integration of advanced equipment, science and technology in Paralympic sports enhances performance and inclusivity, while playing a pivotal role in injury prevention and athletic longevity. Customized solutions can minimize the risk of injury by ensuring better fit and functionality and reducing physical load strain in both Winter and Summer Para sports (65–67). As the field of adapted equipment and technology continues to evolve, its integration with training monitoring (68–70). further amplifies its role in mitigating injury risks and ensuring the well-being of athletes. This, in turn, supports the sustainability and continued growth of Para sports.

## Medication interventions

Medication use in Paralympic sport must be interpreted within the broader context of health–performance interactions. While many medications are prescribed to manage underlying conditions or secondary symptoms, their effects on alertness, neuromuscular function, and exercise tolerance may directly influence training quality and competitive performance. Consequently, health management in Paralympic sport involves continuous decision-making processes that balance symptom control, athlete safety, and performance demands.

There are two main categories of substances to consider: those banned by the World Anti-Doping Agency (WADA) for improving performance or being masking effects, and those are not banned, such as nutritional supplements. Knowledge of the banned list is important so that the athlete and health team are not punished for knowingly or unknowingly using/prescribing the substance. For example, in 2013, Japanese athletes tested positive for the use of glycerol, which can be a component of a lubricant for self-catheterization as a masking agent and is therefore prohibited (71). This has led to adjustments in the standards for urine tests and changes in athletes' routines (72).

In Para sports, a highly debated issue concerns the prevention of boosting among athletes with spinal cord injuries, who may improve their performance through the induction of autonomic dysreflexia. Blood pressure control tests are conducted on athletes before competitions to avoid this practice, and the threshold for a positive test is >160 mmHg (72).

On the other hand, the use of drugs that interfere with sports functionality has been discussed, such as the use of botulinum in athletes with cerebral palsy, in order to increase the range of motion and reduce painful spasticity, which can balance the movement pattern and improve performance. However, the substance is not banned by the WADA (72).

Another relevant aspect concerns the medications prescribed to treat athletes' underlying conditions, which can affect their competitive performance. Anti-spastic medications, commonly used to control spasticity, may induce sedation and reduce muscle function. This effect can diminish the ability of athletes with spinal cord injuries (73) and those with coordination impairment (74) to engage in vigorous physical exercise or training sessions.

Similarly, anticonvulsants prescribed to people with intellectual impairment or coordination impairments cause sleepiness and vertigo and interfere with muscle contraction patterns (75). Antidepressants and antipsychotics, which have a high incidence of use in athletes with intellectual disabilities, have positive and negative influences on physical performance (76). Finally, it is worth noting that drugs like anti-inflammatories and analgesics negatively impact (i.e., reduce) adaptation to training and sport-specific performance (77).

Thus, various aspects need to be considered from an interdisciplinary perspective when organizing the training and competition in the realm of Paralympic sports. The exchange of information among practitioners and sports scientists will be crucial for understanding how athletes' performance may be positively or negatively affected during phases of medication changes and dosage adjustments.

## Conclusions

The Paralympic sports context is complex, and acquiring knowledge is essential to enhance the physical and technical performance of individuals with disabilities. The progression of studies mirrors the achievements of Para athletes, underscoring the increasing need for technical and scientific expertise in the pursuit of excellence. This athlete development process, grounded in a biopsychosocial approach and supported by advancements in applying psychobiological knowledge, is crucial. It facilitates understanding of the effects of training, technological innovations, and modifications to the competitive environment. In the coming decade, new challenges are emerging, with the most significant being the universalization of knowledge. This goal is designed not only to support Paralympic athletes in their pursuit of medals, with the assistance of coaches and multidisciplinary teams, but also to facilitate opportunities for children and young people with disabilities. for inclusion, skill development, and citizenship through the knowledge created and refined in high-performance sport.
